# Centered on child health: Zhu Futang and the foundations of pediatrics and measles prevention in China

**DOI:** 10.1093/procel/pwag008

**Published:** 2026-02-16

**Authors:** Huan Liu, Lu Zhang, Qiangyu Xiang, Hao Cheng

**Affiliations:** University of Science and Technology of China, Hefei 230026, China; State Key Laboratory of Virology, Wuhan 430072, China; University of Science and Technology of China, Hefei 230026, China; University of Science and Technology of China, Hefei 230026, China; Institute of Microbiology, Chinese Academy of Sciences, Beijing 100101, China

Zhu Futang (诸福棠, 1899–1994) (Fig. 1) was a pivotal figure in establishing the foundations of pediatrics and measles prevention in China. His academic career began in the mid-1920s and extended into the 1980s, spanning nearly six decades. His research encompassed experimental medicine, pediatric nutrition, infectious disease prevention, immunology, and clinical pediatrics, and was consistently centered on a core question: how to effectively improve child survival and health underresource-constrained settings. Through sustained research and practice, Zhu Futang progressively established the foundations for approaches to pediatric disease prevention and health care that were applicable to real-world conditions. With respect to measles, a long-standing threat to child survival, Zhu Futang integrated experimental research, clinical observation, and public health approaches, thereby contributing to the gradual transition from passive to active immunization and from individual-based prevention and treatment to population-level prevention.

**Figure 1.**
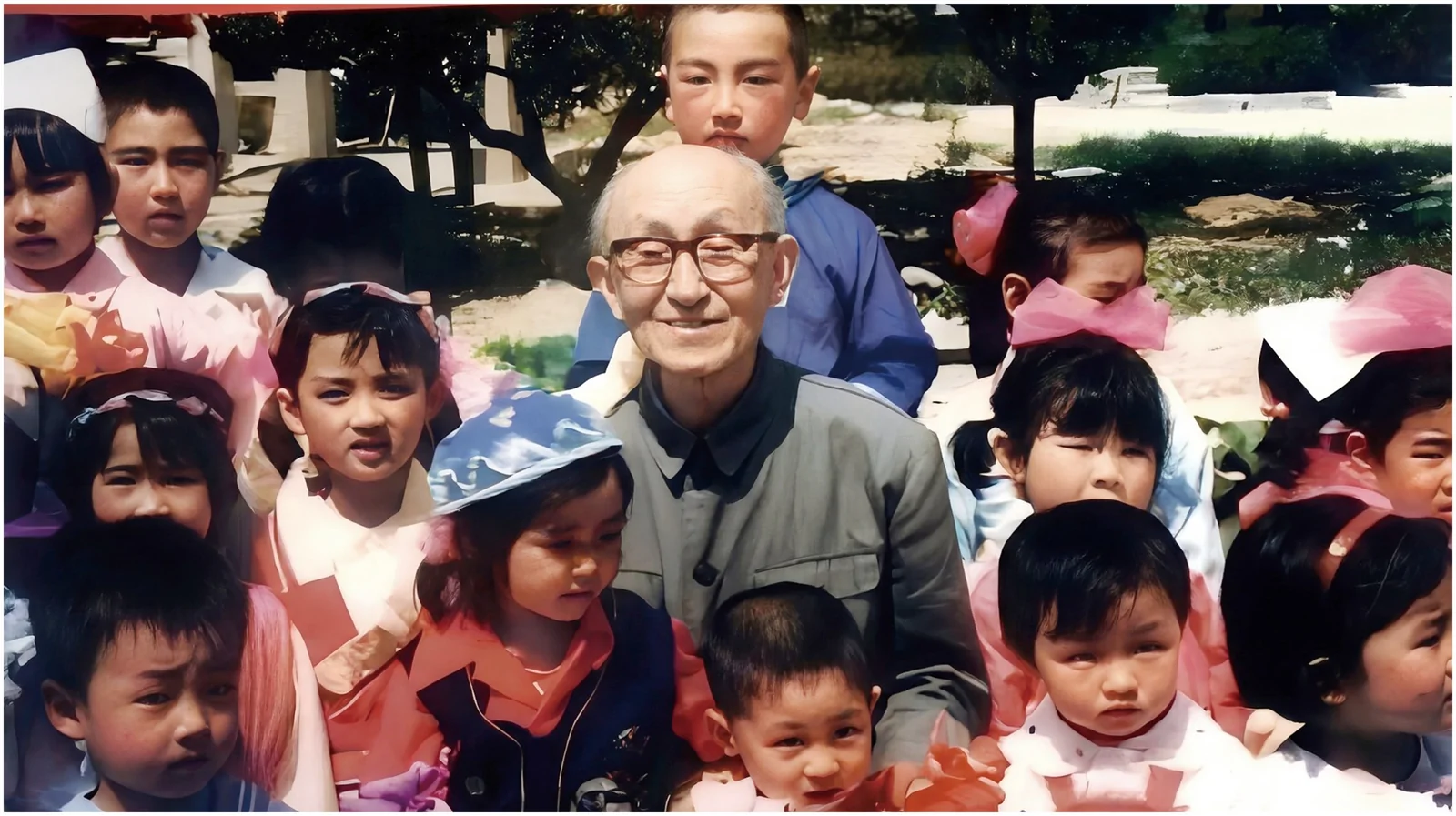
Zhu Futang with children (Source: the CAS Academicians Library).

## Introduction of experimental immunology into pediatric research in China and the advancement of passive measles immunization

Zhu Futang’s academic career was grounded in experimental medicine and systematic clinical observation. In 1926, he published an experimental study on motherwort (*Leonurus sibiricus*), marking one of his earliest scientific publications. This study analyzed the biological effects of traditional medicinal plants using modern experimental methods, reflecting an early emphasis on experimental validation and objective observation rather than reliance on experience-based judgment (Chu and Chen, 1926). In 1929, Zhu Futang conducted an epidemiological analysis of infant tetany among children in Beijing orphanages, examining its distribution by sex, age, and season. Based on a defined population of children in a single institution, he examined the distribution patterns of the disease and feeding practices, contributing to an approach that later emphasized clinical investigation and immunological indicators (Liu and Chu, 1929).

In the early 1920s, Zhu Futang collaborated with the American pediatrician Alexander Ashley Weech. They reviewed four cases of pediatric tuberculosis admitted to Peking Union Medical College Hospital between 1923 and 1927 and analyzed their clinical characteristics. The study found that the patients were predominantly young children and were clinically characterized by extensive pulmonary infiltration, with predominant involvement of the upper lobes. Overall, the patients were in relatively good general condition. Tuberculin testing was frequently positive, whereas sputum smear examinations were predominantly negative. Although the disease course was prolonged, the overall prognosis was favorable, with residual enlargement of hilar lymph nodes often observed (Weech and Chu, 1930). In 1931, Zhu Futang undertook advanced training at Boston Children’s Hospital, affiliated with Harvard Medical School, during which his research focus gradually shifted toward immunology and infant health. In 1933, Zhu Futang and McKhann reported that placental extracts, following appropriate processing, exhibited antibody-like activities in qualitative assays, including neutralization of diphtheria toxin, attenuation of scarlet fever rashes, neutralization of poliovirus, and preventive effects among contacts of measles (McKhann and Chu, 1933). In the same year, in a clinical study focusing on the prevention and attenuation of measles, McKhann and Zhu Futang further clarified the optimal timing of passive immunization (McKhann and Chu, 1933).

During the same period, Zhu Futang investigated infant nutrition, publishing studies on the use of soy milk in infant feeding and conducting systematic observations of feeding regimens based on roasted soybean flour (Chu,1933; Chu et al., 1940). Given the limited material resources and insufficient milk supply at the time, these studies held significant practical relevance and reflected an academic orientation closely aligned with clinical practice and social needs. In the early 1940s, Zhu Futang continued his investigations into the use of placental globulin for measles prevention and disease modification. In addition to examining its role in preventing infection and attenuating disease severity, he sought to standardize dosing through systematic study (Chu and Liu,1944). This work represented a shift from early exploratory applications of passive immunization toward more standardized practice. In the historical context of widespread measles and high childhood mortality, Zhu Futang’s studies contributed practicable approaches to improving pediatric prognosis and reducing the incidence of complications.

## Promoting the development of child public health in China

**Figure 2.**
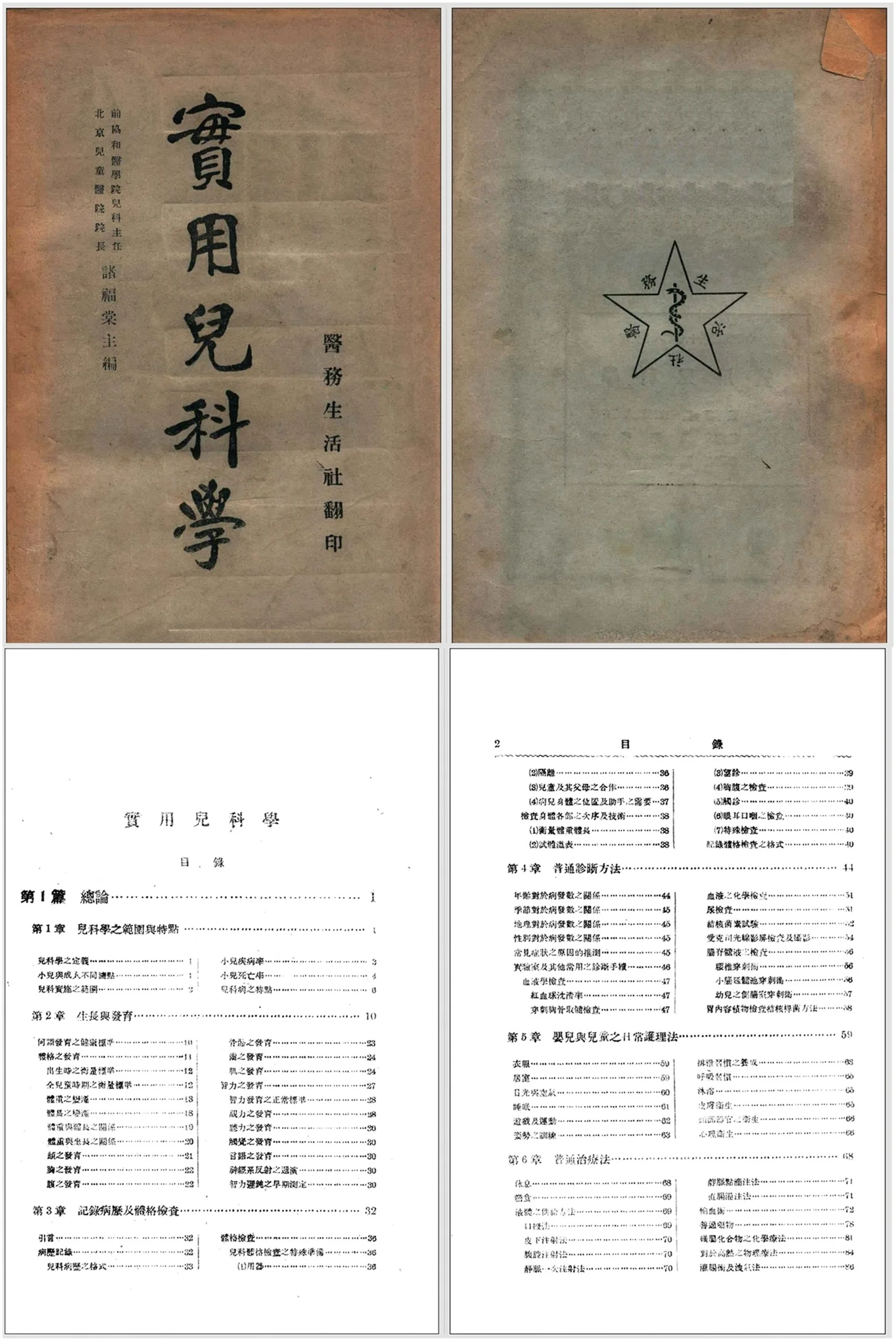
The front and back pages and the table of contents of *Practical Pediatrics*, the first edition.

In 1942, Zhu Futang, together with the pediatric infectious disease specialist Wu Ruiping and the pediatrician Deng Jinqin, founded the North Beijing Private Children’s Hospital in Beijing, the predecessor of Beijing Children’s Hospital. This initiative contributed to the gradual emergence of pediatrics in China as a specialized discipline, distinct from its earlier status as a subspecialty within general hospitals. In 1943, Practical Pediatrics, a comprehensive textbook of approximately 800,000 words, was published. Oriented toward clinical practice, the book covered a wide range of topics, including child growth and development, physical examination and diagnosis, infant feeding and care, neonatal disorders, nutritional disorders, infectious diseases, and parasitic diseases, with conditions organized according to body systems (Fig. 2). Ranging from prevalent infectious diseases of the period, such as measles and diphtheria, to less common endocrine disorders, including diabetes and precocious puberty, the book covered a broad spectrum of pediatric conditions as well as key aspects of growth and development relevant to clinical practice. The book incorporated international research findings and statistical data, while also compiling clinical and research data on Chinese children from the Department of Pediatrics at Peking Union Medical College Hospital and other Chinese investigators. The book has served as an important reference for both academic research and clinical practice. Approximately 80% of the initial manuscript was authored by Zhu Futang, with contributions from Fan Quan, Su Zufei, Wu Ruiping, Deng Jinqin, and other collaborators. This book was among the earliest comprehensive modern monographs on pediatrics in China. Initially edited by Zhu Futang, it was later published under the title *Zhu Futang’s Practice of Pediatrics*. To date, the book has undergone eight revised editions and has long served as a widely used textbook in Chinese pediatrics, playing an important role in the training of generations of pediatricians (Mao and Gu, 2023).

With respect to hospital development and disciplinary advancement, Zhu Futang emphasized both institutional structures and professional ethos. In 1949, drawing on his extensive experience in medical practice and hospital administration, Zhu Futang, together with Wu Ruiping and Deng Jinqin, formulated a hospital motto—“Public Service, Benevolence, Diligence, and Harmony.” Zhu Futang personally inscribed the motto, which was subsequently displayed in the outpatient hall. Since that time, Beijing Children’s Hospital has undergone sustained development with continued governmental and societal support (Hou and Pang, 2011). From the 1950s onward, Zhu Futang served as a member of the Medical Science Committee of the Ministry of Health. He served for many years as Chair of the Pediatrics Branch of the Chinese Medical Association and as Editor-in-Chief of the Chinese Journal of Pediatrics, while also holding concurrent positions as Vice Chair of the People’s Committee for the Defense of Children of China and Director of the Institute of Pediatrics at the Chinese Academy of Medical Sciences. These positions allowed him to be based in Beijing while extending his professional engagement nationwide and maintaining an international perspective. From a broader viewpoint, he could thereby assess the overall development of pediatric care in China.

After the founding of the People’s Republic of China, Zhu Futang began to systematically address the role of pediatrics within the national public health system, gradually expanding his focus from the prevention and treatment of individual diseases to a broader, population-based framework for child health. During the 1950s, he published a series of articles on topics including the diagnosis and management of epidemic encephalitis B, the orientation of pediatric practice, and strategies for the development of child health care. In 1952, in an article addressing the management of epidemic encephalitis B, Zhu Futang systematically outlined isolation measures, mosquito control, nursing care, and specific therapeutic interventions (Chu et al., 1952). In 1958, Zhu Futang argued that child health care and disease prevention should occupy a central place in pediatric practice and emphasized the critical role of health education and mass public health campaigns in the control of infectious diseases. He also advocated the study and promotion of a community-based health care system to guide health practices within families and kindergartens (Chu, 1958). In 1959, Zhu Futang conducted a systematic review of a decade of progress in child health care, using Beijing as a case study. The review examined the development of children’s hospitals, the expansion of pediatric services at the district level, advances in early childhood education, and the integration of local health services with patriotic public health campaigns (Chu, 1959). Subsequently, he expanded his analysis to the national level, addressing progress in areas including the control of major infectious diseases, reductions in child mortality, the establishment of pediatric research institutions, the development of child care services, and the training of pediatric professionals (Chu, 1959). Through this body of research and synthesis, Zhu Futang contributed to the transformation of pediatrics in China from a primarily clinical specialty into an integral component of the child health care system.

## Advancement of active measles immunization and the early application of live attenuated vaccines in China

In the early 1960s, attenuated live measles vaccines were rapidly developed and introduced internationally. Zhu Futang recognized this international trend and organized and led related research and practical efforts in China. He collaborated with specialists in virology, biological products, and child health from major centers including Beijing, Shanghai, and Changchun. Using an attenuated live measles vaccine independently developed in China, they conducted vaccination programs in collective child-care settings, including nurseries, kindergartens, and primary schools, and systematically assessed clinical responses, immunologic outcomes, and epidemiological impact. This work yielded positive outcomes, and its findings were first implemented in urban Beijing, providing a basis for the broader introduction of childhood measles vaccination and contributing to the subsequent reduction of measles outbreaks.

With the deepening of research on attenuated live vaccines, Zhu Futang continuously participated in and promoted related clinical and immunological studies, conducting a systematic evaluation of the safety and immune response of the vaccine in the pediatric population. In 1962, Zhu Futang used human amniotic cells to propagate attenuated measles virus as the subject, comparing the clinical and immunological responses of two different passage backgrounds of the virus strains and different infection doses in children, proving that the attenuated live measles vaccine using human amniotic cells was acceptable in terms of safety and had good immunogenicity in children, laying an important foundation for the scientific and standardized implementation of the active immunization strategy for measles in China, and being one of the key academic achievements of Zhu Futang’s transition from passive immunity to the era of vaccines in the research on measles prevention and control (Huang et al., 1962).

**Figure 3.**
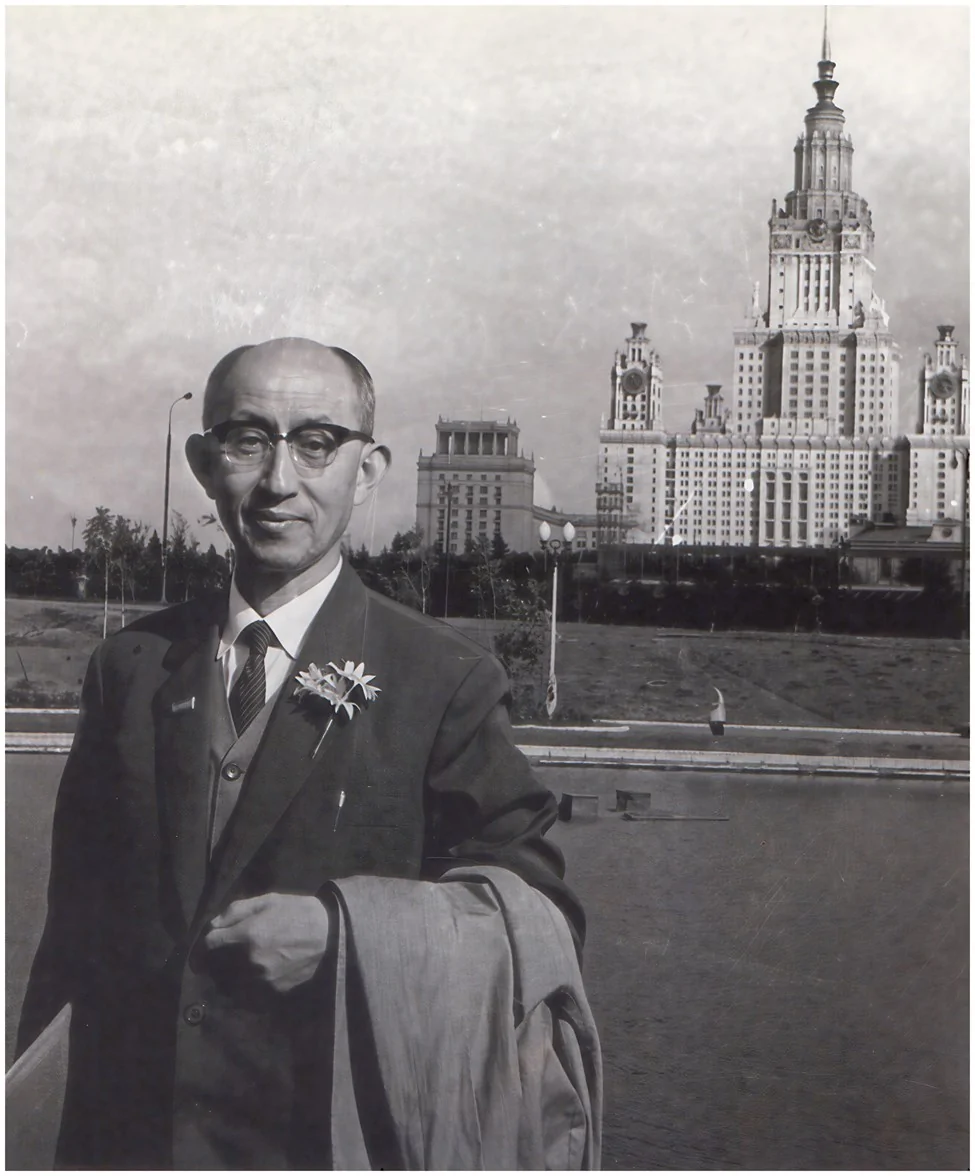
In 1963, Zhu Futang attended the Soviet Union Conference of Pediatrics at Moscow.

In 1963, Zhu Futang traveled to Moscow to attend the All-Union Congress of Pediatrics, reflecting China’s active engagement in international pediatric exchange during a critical period of child health system development (Fig. 3). In 1964, Zhu Futang outlined four major international approaches to measles vaccine research: the use of highly attenuated live vaccines alone; combined administration of partially attenuated live vaccines with gamma globulin or placental globulin; vaccination of infants who retained maternal measles antibodies; and the use of inactivated vaccines alone or prior to attenuated live vaccination. He further argued that China should pursue progress across all four approaches and noted that advances had already been made in this regard. This work represented a turning point in measles prevention and control in China, reflecting a shift from reliance on passive immunization and clinical treatment toward strategies centered on active immunization and population-based prevention (Chu et al., 1964). In the same year, in discussions concerning the use of antibiotics in the early stage of measles, he emphasized the importance of preventive immunization among susceptible individuals and suggested that the optimal timing was the fifth to sixth day following initial exposure. This timing was considered to correspond with the exposure history of most children and to facilitate attenuation of disease severity. Through a systematic assessment of the benefits and limitations of prophylactic antibiotic use, Zhu Futang articulated an early measles prevention and management strategy centered on passive immunization and nursing care, with medication guided by clinical indications. This work represents a key expression of his thinking on the public health–oriented prevention and control of measles (Chu, 1964). In 1965, in an article with a practical focus on measles prevention and control, Zhu Futang provided explicit dosage recommendations, including placental globulin at doses of 3–6 mL and parental blood at 20–30 mL. He emphasized that inadequate dosing could limit preventive efficacy and reduce the attenuation of complications. By systematically clarifying the identification of susceptible populations, principles of nursing care, isolation measures, and strategies for passive immunization, this research articulated a measles prevention and control framework oriented toward primary-level health settings and kindergartens. It represents a developed expression of Zhu Futang’s public health–oriented approach to measles prevention (Chu, 1965).

## Academic legacy and sustained influence in pediatric centered on child health

Following China’s reform and opening-up, Zhu Futang increasingly devoted his efforts to the advancement of pediatrics as an academic discipline, participating in research activities primarily in senior advisory and intellectual leadership capacities. He promoted the overseas training of young scholars and facilitated opportunities for international conferences and academic exchanges for pediatric experts from various regions of China, thereby fostering broader scholarly exchange and international engagement.

**Figure 4.**
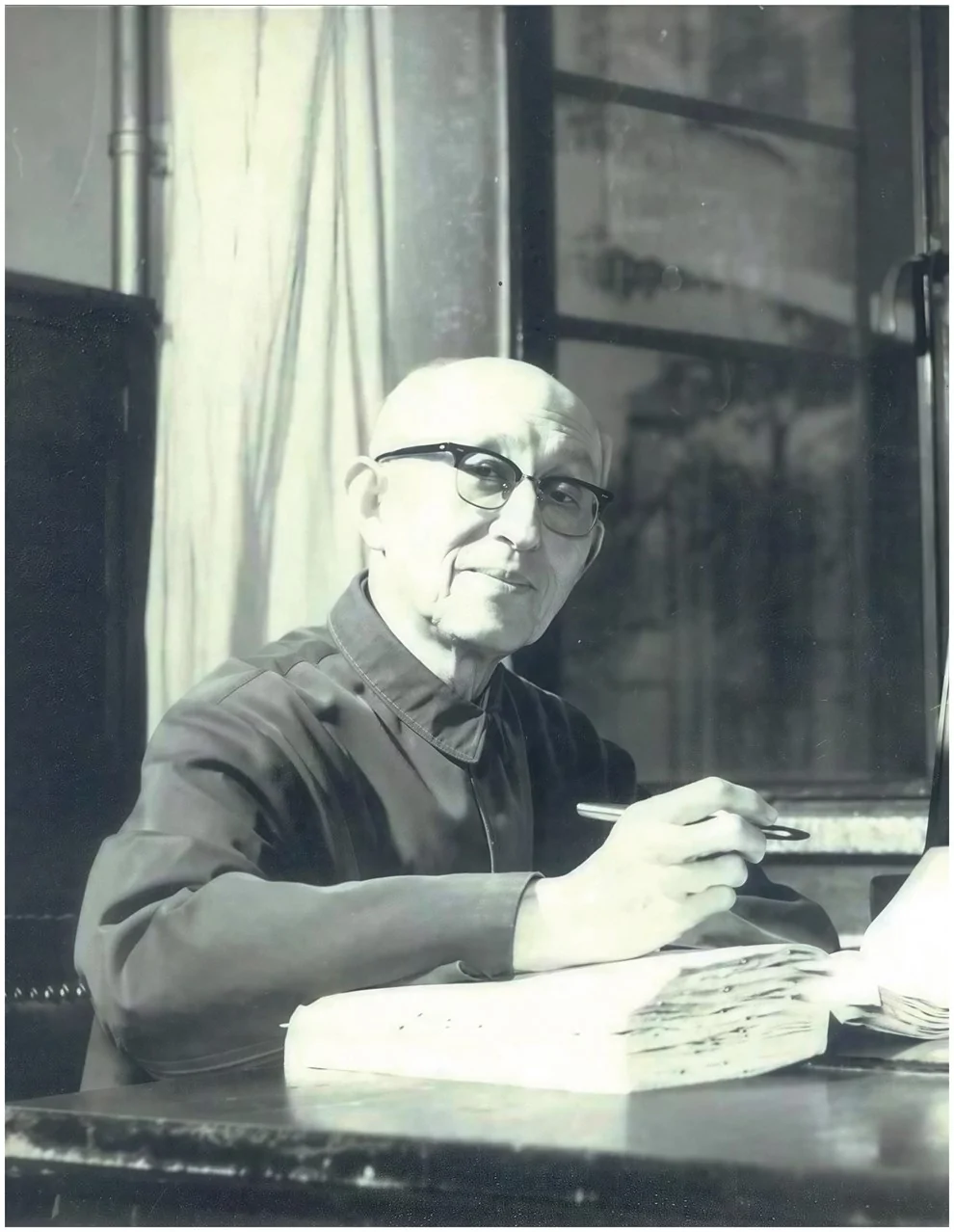
Zhu Futang in the office.

In his later years, Zhu Futang continued to engage in professional work and academic pursuits, while providing outpatient services and conducting ward rounds (Fig. 4). In 1982, Zhu Futang resigned from his positions as President of Beijing Children’s Hospital and Chair of the Pediatrics Committee of the Chinese Medical Association. He argued that, as the pediatric workforce became increasingly well established, the discipline should adhere to what he termed the principle of “generational succession,” thereby promoting intergenerational succession and coordinated development. In 1984, researchers at Beijing Children’s Hospital, under the supervision of Zhu Futang, published a study comparing blood and cerebrospinal fluid lactate levels in children with severe pneumonia and respiratory failure with those in a control group. The study found that blood and cerebrospinal fluid lactate levels were significantly elevated in children with respiratory failure and that the two measures were positively correlated. These findings suggest that, under conditions of severe hypoxia and metabolic disturbance, metabolic changes in the central nervous system may be reflected by cerebrospinal fluid biomarkers (Yang et al., 1984). In 1985, the research team further established the relationship between blood and cerebrospinal fluid acid–base parameters in healthy children, standardized sampling and measurement procedures, and provided a foundational reference for determining whether changes in cerebrospinal fluid acid–base status are solely attributable to alterations in blood gas levels (Yang et al., 1985). In the same year, a study examining changes in cerebrospinal fluid acid–base parameters during respiratory failure adopted a continuous observational approach to assess alterations in blood–cerebrospinal fluid acid–base balance (Yang et al., 1985). Although Zhu Futang was no longer directly involved in the primary experimental work at this stage, his academic judgment, approach to research design, and clinical perspective continued to shape the direction of the studies and the interpretation of the results. This phase of work reflected his role in mentoring successive researchers and guiding research practices, contributing to the continuity of his academic approach.

**Figure 5.**
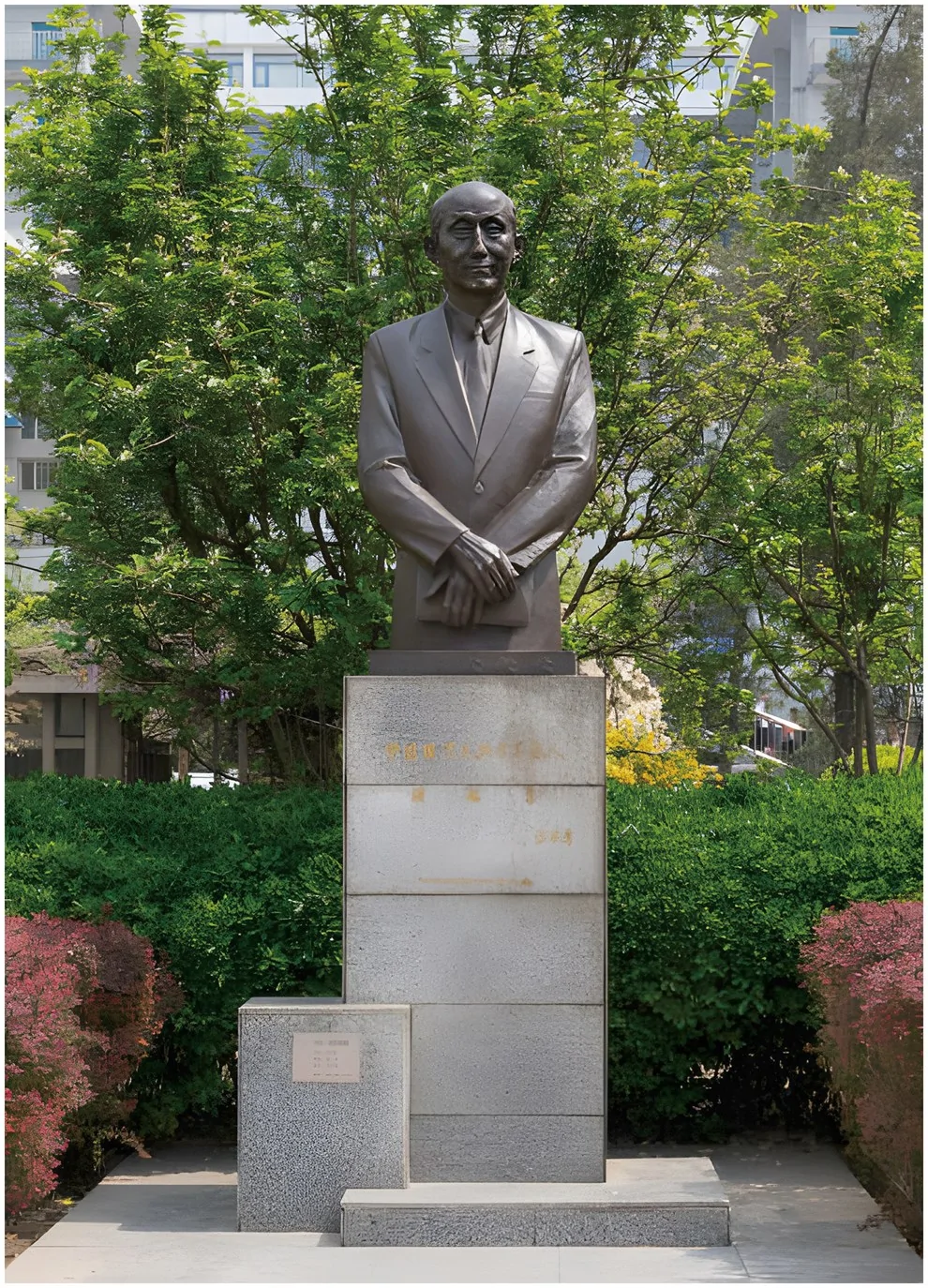
The bronze statue of Zhu Futang in the West Garden of Beijing Children’s Hospital (Built in 1992).

In 1991, the Ministry of Health of China, in collaboration with the Canada-China Children’s Health Foundation, established the Zhu Futang Award in his honor to recognize pediatricians who have made outstanding contributions to pediatric clinical practice, scientific research, and child health care in China. In 1992, Li Shouren commissioned a bronze statue in memory of Zhu Futang. The statue is currently located at Beijing Children’s Hospital and symbolizes his academic spirit and historical contributions (Fig. 5). Zhu Futang passed away on April 23, 1994, at the age of 94, concluding a career devoted to pediatric medicine and child health. A review of his academic career highlights an approach that integrated scientific research, clinical practice, and a commitment to public health. This integrative perspective continues to inform contemporary pediatric medicine and child health research.

## Data Availability

All data used in this study are available in the published literature.

## Abbreviations

CAS: Chinese Academy of Sciences
